# Adverse Psychosocial Work Environments and Depression–A Narrative Review of Selected Theoretical Models

**DOI:** 10.3389/fpsyt.2020.00066

**Published:** 2020-02-27

**Authors:** Johannes Siegrist, Natalia Wege

**Affiliations:** ^1^ Centre for Health and Society, Heinrich-Heine University Düsseldorf, Düsseldorf, Germany; ^2^ Clinic for Psychiatry and Psychotherapy, Heinrich-Heine University Düsseldorf, Düsseldorf, Germany

**Keywords:** psychosocial work environment, depression, scientific evidence, demand-control, effort-reward imbalance, organizational injustice

## Abstract

Far-reaching progress of treatment and prevention of depressive disorders is still limited, mainly due to the multifactorial determinants of these disorders and the restricted knowledge of their aetiology. Stressful socio-environmental conditions represent one of the multifactorial determinants, and in view of the centrality of work and employment for human well-being, research on health-adverse psychosocial work environments turned out to be a promising line of scientific inquiry. During the past three decades, respective research focused mainly on three theoretical models of adverse psychosocial work and their measurement in prospective epidemiologic studies, termed “demand-control,” “effort-reward imbalance,” and “organizational injustice.” This report provides a review of current evidence on their associations with depression, based on several systematic reviews and updated by most recent publications. Moreover, it discusses the conceptual and methodological strengths and weaknesses of these associations. In summary, the results of more than 40 cohort studies from a variety of Western modern societies confirm that stressful work in terms of these models is associated with a moderately increased risk of subsequent onset of depression. While this knowledge is considered robust enough to instruct efforts of primary and secondary prevention, several methodological challenges still need to be resolved by future research.

## Introduction

In a global perspective, depressive disorders are a leading cause of years of life lost to disability (1). Due to their prevalence, severity, and associated direct and indirect costs, these disorders provide a major challenge to health care systems (2). Although the definition and classification of depression continues to be debated (3) epidemiologic studies offer solid estimates of their prevalence, at least in modern Western societies. For instance, in the USA, the 12-month prevalence of a major depressive episode was estimated as 6.6% (4). A similar rate of 6.9% was observed in a study of 17 European countries (5). Given the public health relevance of depression, efforts of prevention and intervention are required. Yet, as this disease seems to be caused by an interaction of genetic, biological, psychological, and socio-environmental conditions (6), the success of such efforts is still limited, and intense research continues to tackle this problem. One promising line of recent research has focused on an adverse psychosocial work environment in advanced economies as a social determinant of depression. This research relies on distinct theoretical concepts derived from social and behavioral sciences and their standardized measurement, and it generates its findings in the frame of prospective epidemiologic investigations. During the past three decades, a considerable body of scientific knowledge resulted from this approach. Therefore, a review of its conceptual and methodological strengths and limitations seems justified. Here, we set out to meet this aim.

## Stress-Theoretical Approaches and Empirical Evidence

As depression is a mental disorder, it was proposed that altered functioning of distinct brain areas and related neurotransmitter release is involved in its development (7). More specifically, stress-physiological mechanisms elicited by the brain reward system and operating through a dysregulation of neurotransmitter and hormone release (e.g., serotonin and cortisol) may contribute to the manifestation of biological, affective, cognitive, and behavioral symptoms of this disorder (8, 9). To strengthen this hypothesis, it is important to delineate those socio-environmental conditions that are associated with increased risks of incident depression, acting as extrinsic stressors that trigger the proposed psychobiological mechanisms. Extrinsic stressors act as single or recurrent challenges that tax or exceed the person’s coping capacities, thus inducing intense experiences of threat or loss of control and reward related to core desiderata. In modern working life, several such threats and losses are widely prevalent, and given the centrality of work and employment for people’s self and their social standing, they are likely to affect their functioning and wellbeing. Yet, an important question remains to be answered: How can these extrinsic stressors at work be identified? To this end, the development and test of a theoretical model derived from social and behavioral sciences is required.

### Three Theoretical Models of a Health-Adverse Psychosocial Work Environment

A theoretical model in this domain delineates selective features of a demanding and threatening work environment such that they can be generalized to explain associations of stressful work with health in a wide range of occupations. Given their selective focus, several such theoretical concepts were proposed [for review, e.g., (10)]. Yet, three approaches received prominence in recent years, as documented in a substantial number of empirical studies performed in occupational epidemiology, sociology and psychology.

The first approach is termed “*demand-control* (or *job strain*) model” (11). It posits that stressful experience at work results from exposure to a distinct job task profile defined by the combination of two dimensions, the psychological demands put on the working person, and the degree of control available to perform the required task. Jobs with high demand and low control are stressful because they limit the individual’s autonomy and sense of control while generating continued pressure (high strain). In this model, low control manifests itself as a lack of decision authority and/or as a lack of opportunity to use one’s skills (e.g., monotonous work). A further distinction points to the role of social support at work. If people exposed to high demand and low control at work additionally suffer from social isolation and lack of social support, the level of stress is further increased (12). So far, this model has received its broadest implementation on a global scale, and has generated a large amount of evidence (see below). At the same time, this conceptualization was developed during a stage of economic development where industrial production prevailed, with inherent forms of division of labor in hierarchically structured organizations. Additional concepts may address more recent developments of work and employment.

“*Effort-reward imbalance*” is one such complementary theoretical model focusing on a basic notion of the work contract, the norm of social reciprocity. It maintains that a lack of reciprocity in terms of high effort spent at work by employed people, and low reward provided in turn by employers, acts as an extrinsic stressor. In this exchange, three basic types of reward are transmitted: salary or wage, career promotion and job security, and esteem or recognition (13). According to this approach, failed reciprocity at work occurs frequently in modern labor markets, given a growth of insecure and precarious employment, short-term contracts, and new forms of flexible job arrangements. Moreover, rising income inequality and a large proportion of working poor point to the relevance of this notion. Effort-reward imbalance is frequent if workers have no alternative choice in the labor market and if jobs are characterized by heavy competition. Moreover, this model integrates an important element of the working person, the pattern of coping with extrinsic obligations. Overcommitted people are at elevated risk of experiencing this imbalance and its health-adverse effects (14).

As a third model, “*organizational injustice*” is dealing with perceived inequities of people’s behaviors in formal organizations. It was developed in the context of organizational psychology and management sciences in the 1970s and 1980s, being largely based on Adam’s inequity theory (15, 16). Four types of injustice are usually distinguished. Procedural injustice points to the perceived deviance from established rules of decision-making and of judging the performance of employees. Relational or interactional injustice describes the unequal treatment of persons within organizations, e.g., with regard to respect and communication. With informational injustice the unequal access to and share of relevant information within organizations is emphasized. Finally, distributive injustice delineates the perceived inequity of an organization’s distribution of valuable goods, resources, and services to its members. According to this approach, each type of inequity can evoke stressful experience, and thus act as health-adverse psychosocial work environment (17).

Although a minor overlap between these concepts can be observed (e.g., between “demand” and “effort”; between “reward” addressing intrapersonal justice of exchange and “distributive injustice,” addressing interpersonal inequity), each theoretical model has identified a distinct psychological need whose fulfillment is essential for personal flourishing and well-being, and whose suppression results in emotional suffering and recurrent arousal of stress responses. The experience of personal control and self-efficacy in productive activities describes the focal element in the first model. In the second model, the experience of appreciation and self-esteem emanating from one’s achievements is the core element. Experiencing recurrent trust, fairness, and a sense of belonging within a stable social network is central to the third model. What happens to mental health and well-being if these crucial needs remain unmet, or are even denied in everyday working life? Before we turn to this question, important methodological problems need to be addressed.

### Methodological Challenges

The first challenge of this research concerns the measurement of the theoretical models and of the health outcome, i.e., depression. These two tasks differ to some extent, as the assessment of depression is part of clinical decision making, whereas the measurement of a theoretical construct originating in the social and behavioral sciences usually relies on quantitative research methods, and more specifically on psychometrically validated scales of self-assessed questionnaires. These scales operationalize the single dimensions of the construct by a set of standardized items. While there are different response options to the items, Likert-scaled items are most often applied, where answers range, e.g., from “strongly disagree” to “strongly agree,” with varying number of answer categories. Sum scores of the ratings of each scale are assumed to represent a quantitative estimate of the dimension under study. Scale development is a first fundamental step of assessing a theoretical model. Yet, each single scale represents one factor only of a more complex construct. Therefore, in a second step, the dimensional structure of the model has to be examined, using confirmatory factor analysis. The results of this analysis indicate how well the combination of the single factors represents the underlying concept. Structural equation modeling is an appropriate statistical approach allowing an assessment of the goodness of model fit, i.e., the degree of congruence between observed data and postulated theoretical structure.

This second step is not trivial. For instance, in case of the demand-control model, different results are obtained, depending on whether the two main scales, “demand” and “control” are included, or whether the third variable “social support” is additionally examined (18). The effort-reward imbalance model offers an even more complex structure as it is composed by a first-order construct representing the three scales “effort,” “reward,” and “overcommitment,” and a second-order construct including the three subdimensions of “reward,” “esteem,” “job security,” and “job promotion prospects” (19). In this latter case, the goodness of the model fit based on the second-order construct should be better than the one based on the first-order construct. This has been repeatedly documented (20).

Each review of studies testing the contribution of these theoretical models towards explaining elevated risks of depression is faced with the problem that in some studies, findings are restricted to single model scales, whereas other investigations use a summary measure of the model to estimate its explanatory contribution. For instance, with regard to the demand-control model, several publications provide results for the scale “job control,” rather than for a combined measure representing the joint effect of demand and control, termed “job strain.” Similarly, in case of “organizational injustice,” results indicate associations with single dimensions rather than with an overall measure of the model (21) (see below). This fact compromises the comparability of study findings. In addition, it points to the statistical problem of representing a multifactorial concept by a single summary indicator, such as “job strain” (a combination of the scales “demand” and “control”) or “effort-reward imbalance,” a ratio of the scales “effort” and “reward,” adjusted for unequal number of items (see Discussion).

As in every scientific discipline, the reliability and validity of data represent crucial quality criteria of reported findings. Whereas researchers examine the reliability of questionnaire data by established procedures, the validity of self-reported information is often challenged, given a lack of objective standard of reference. Several strategies were developed to deal with this problem. To mention just three common strategies, the first one refers to the control of reporting bias due to distinct personality characteristics by respective statistical adjustment. A second approach uses triangulation of subjective data with objective information (e.g., by comparing self-reported data of participants with observer-based or administrative data. Third, individual data are aggregated to the group level to reduce variability of subjective evaluations (e.g., by applying mean scale scores at work-unit level rather than individual scale scores as predicting variables). In fact, the validity of respective results has been improved by applying these strategies [e.g., (22, 23)]. However, one should recognize that the working people’s own experience is a core source of information in any research dealing with psychosocial exposures and their psychological and biological effects.

The problem of validity concerns the measurement of depression, the outcome criterion, as well. In psychiatry, the Composite International Diagnostic Interview (CIDI) is considered a well- tested approach to diagnose clinically relevant depression (usually termed “major depression”) (24). Yet, in epidemiologic studies of large cohorts, clinical assessments are often not feasible, and are replaced by validated questionnaires. The Centre for Epidemiologic Studies Depression Scale (CES-D) is one such widely applied questionnaire, among several others (25, 26). These scales assess depressive symptoms rather than a binary variable of clinical depression, and they equally have to deal with the challenge of limited validity as they use self-reported data from study participants. There are pros and cons of using questionnaire data on depression. On the positive side, large-scale information offers robust estimates of disease incidence, and assessing depressive symptoms rather than manifest clinical depression enables researchers to identify the burden of subclinical mental disorder, which is relevant in a public health perspective (27). In addition to psychiatric interviews and questionnaires, administrative data are used to identify people with depression, e.g., based on records from health insurance or from hospitals. Again, any review of available evidence faces the problem of limited comparison of study results, given the different ways of operationalizing depression or depressive symptoms.

An important further methodological challenge relates to the study design. Prospective observational studies of large cohorts of working people are the best available approach, whenever experimental designs are not feasible to assess a causal relationship. In epidemiologic cohort studies, the exposure (in our case, work stress) is assessed prior to the incidence of the disease (in our case, depression), and a dose-response relationship between level of exposure and strength of its association with disease risk can be examined, with regression adjustment for confounders in multivariable statistical analysis. Yet, support of the notion of a causal association in epidemiologic studies depends on additional criteria. They include the consistency of findings by recurrent independent replication, the provision of biological data or other mediating information that substantiate some pathway leading from exposure to disease development. Moreover, reducing exposure through intervention is expected to minimize subsequent disease risk. Below, we will discuss to what extent research on the three theoretical models and their association with depression meets these methodological challenges.

### Summary of Study Findings

The aim of this contribution is to provide a summary of main findings on associations of an adverse psychosocial work environment, as measured by three prominent theoretical models, with depression, and to discuss the conceptual and methodological strengths and limitations of this knowledge. To this end, published systematic reviews and meta-analyses are a major source of reference. Here, we include the following systematic reviews: (21, 26, 28–30). Information derived from these reviews is supplemented by results from publications that were published after the appearance of these reviews.

#### The Demand-Control Model

In addition to four previously published reports (31–33), two recent systematic reviews of associations between job strain (or the components “demand” and “control”) and depression provide a solid empirical basis of assessing this relationship, one from Sweden (26) and one from Denmark (28). These latter reviews not only update the evidence-base, but they also apply quality assessments of the included studies, and, in one case, integrate the findings from a series of unpublished studies. We therefore focus our report on these recent reviews, supplementing them, where needed, with previous and more recent findings. The two reviews differ in several regards. First, the study by Theorell et al. (26) includes a broader range of exposures (different psychosocial work stress models, physical and chemical work stressors), while Madsen et al. (28) restrict their analysis to the job strain model. Second, the latter report focuses on clinical depression, whereas the former refers to questionnaire-based data on depressive symptoms or interview data. Third, in the Danish study, estimated relative risks of the association are based on adjustments for core sociodemographic variables and for baseline depressive symptoms, thus offering a more robust confounder control.

In the Theorell et al. (26) review, prospective cohort studies published between 1990 and June 2013 were included, where the exposure was defined in terms of job strain in 14 studies, in terms of psychological demands in ten studies, and in terms of job control in 19 studies. In addition, social support at work was assessed in 17 studies, although this variable is not included in the theoretically core construct of job strain. In a majority of cases, job strain was defined by the procedure of median split half of scores of the two scales “demand” and “control,” identifying the exposure group as experiencing “high demand and low control” at work. The Danish review was based on a protocol published before the start of data analysis, thus reducing reporting bias. The protocol described the measurement of core variables and confounders. Original or proxy measures of job strain and its subscales were measured in a similar way across the published and unpublished study, using the split half approach mentioned in a majority of cases. Some exceptions were the use of the quadrant of the combinations of demand and control and the interaction test of the two subscales. Depression was assessed by clinical data in the six published studies and by administrative data on hospital-treated depression in the 14 unpublished investigations included in the review (28). In the meta-review by Harvey et al. (30), no additional relevant findings were identified.

The main findings of the Swedish systematic review and meta-analysis are summarized as follows: Job strain, assessed in 14 studies, was associated with a weighted odds ratio of 1.74 [95% Confidence Intervals (CI) 1.54; 1.96] of depressive symptoms, compared to the group without this exposure. High control at work, as a protective factor, was associated with a weighted odds ratio of 0.73 (95% CI 0.68; 0.77) of incident depressive symptoms in the 19 reports included, compared to those with low control at work. Both findings resulted from studies whose quality was rated as high. However, findings on a link between high demand and depression were inconsistent and generally of lower quality. It is of interest to note that low support at work as well as passive job (low demand and low control) were also related to an increased risk of depression (26).

In the Danish systematic review and meta-analysis, results were given separately for published and unpublished studies. For the first group, the adjusted odds ratio of clinical depression due to job strain was 1.77 (95% CI 1.47; 2.13) compared to those without job strain. This estimate was virtually unchanged if high quality studies only entered the analysis. Interestingly, two investigations reported two subsequent exposure assessments, and in line with the assumption of a dose-response relationship the relatively highest risk was observed in the group with two subsequent work stress exposures [odds ratio (OR) 1.56 (95% CI 0.99; 2.45]. In the unpublished studies, a hazard ratio (HR) of 1.27 (95% CI 1.04; 1.55) was observed, based on a prevalence of job strain of 16.6% (28). Alternative formulations of the demand-control model did not reveal consistent findings, except for the quadrant defining “passive job,” which was related to elevated risk of depression.

In summary, the two reviews confirm previous evidence that job strain is associated with a moderately increased risk of depression, and the subscale of low job control plays a decisive role in these associations. Yet, in a recent critical appraisal it was argued that possible bias due to reverse causation and residual confounding was not convincingly addressed so far (34). Therefore, these authors contributed own findings from a new study, the UK National Child Development Study, where the risk of incident common mental disorder (not specifically depression) over a 5-year observation period was analyzed according to job strain, assessed in a group of 6.870 men and women at age 45. To control for the bias mentioned, a number of additional confounding factors, specifically from childhood, were included, and lifetime psychiatric history was assessed to minimize reverse causation. The findings demonstrated significantly elevated odds ratios for job strain [OR 2.22 (95% CI 1.59; 3.09)], high demand [OR 1.70 (95% CI 1.25; 2.32)], and low control [OR 1.89 (95% CI 1.29; 2.77)]. Thus, we can conclude that job strain is a validated predictor of a moderately elevated risk of developing relevant depressive symptoms or manifest clinical depression.

#### The Effort-Reward Imbalance Model

Compared with the demand-control model, fewer studies were conducted so far using the complementary effort-reward imbalance model of stressful work. A recent review identified eight eligible cohort studies, encompassing 84.963 persons and 2.897 new cases of depressive disorders (29). Previous reviews were based on a lower number of studies, and due to the paucity of findings, the evidence supporting a positive relationship between effort-reward imbalance and depression was judged to be limited (26, 31, 32, 35). The new systematic review was preceded by a published study protocol. To be included studies had to provide a quantitative baseline assessment of the exposure, effort-reward imbalance (ERI), using either the original instrument (19) or a proxy measure. This information was reported as a binary variable (effort-reward ratio yes/no), as a categorical variable, or as a continuous variable. Depression was measured by psychiatric diagnostic interview, physician-based diagnosis, register data, or validated questionnaire (29). In one study, the outcome was defined by register data on purchased antidepressants (36), although the choice of this indicator received serious criticism (37). The results indicated that in seven out of eight studies ERI predicted depressive disorders, with estimates ranging from 1.49 (95% CI 1.22; 1.81) to 2.32 (95% CI 1.14; 4.73), if groups with the highest exposure to ERI were compared to respective reference groups. The study defining depression by use of antidepressants showed no association. When the eight most-adjusted study-specific estimates were pooled in a random-effects meta-analysis, an odds ratio of 1.49 (95% CI 1.23; 1.80) was observed (29). In two studies, a dose-response association was obvious, and one investigation demonstrated similar associations, independent of whether ERI scores were aggregated to the work-unit level or were analyzed at individual level. Results did not change when fixed- rather than random-effects meta-analysis was performed. Moreover, pooled estimates were similar when comparing studies with high or moderate quality vs. studies with low quality, or when comparing studies using the original ERI measure vs. those applying proxy measures. The study authors discussed the controversial use of purchased antidepressants as indicator of depression and, due to this problematic measure, repeated sensitivity analyses by excluding this study with negative results. After exclusion, pooled estimates were all in the range of 1.56 to 1.66 (29).

To summarize, this systematic review of eight prospective studies from Europe, Canada, and the US demonstrated that ERI was associated with a 1.5-fold increased risk of depressive disorders. This conclusion is supported by the meta-review of Harvey et al. (30) although based on a less comprehensive number of studies. Since the publication of the Rugulies et al. review, three new prospective studies on this association were published. In the first investigation from Japan, an odds ratio of 1.56 (95% CI 1.25; 1.96) of depression due to effort-reward imbalance at work was documented (38). In the second one, a large panel study from Germany, gender-specific estimates of work stress in terms of ERI were performed to assess risks of incident doctor-diagnosed depression during a 2-year follow-up period (39). Although work stress levels were higher among men, the risk ratios (RR) of depression due to ERI were similar across gender, with an adjusted RR of 1.88 (95% CI 1.51; 2.33) in women, and of 1.82 (95% CI 1.36; 2.44) in men. In this sample of 6.693 participants estimates were adjusted for age, marital status, education, income, employment, smoking, alcohol consumption, physical activity, BMI at baseline, and any chronic disease at baseline. It is of interest to note that in addition to this summary measure of the effort-reward ratio, significant associations were observed for all single components of the ERI model (39). A recent study on Swedish national panel survey data analyzed bidirectional relationships between psychosocial work characteristics and depressive symptoms with fixed effects (40). Whereas no evidence for a reverse causation from depression to psychosocial work stress was found, high effort at work was prospectively associated with depressive symptoms 2 years later. The remaining dimensions of the three work stress models included displayed short-term associations only.

In line with the conclusion drawn from the meta-analyses based on the job strain model, we can state that effort-reward imbalance at work is associated with a moderately increased risk of developing a depressive disorder.

#### The Organizational Injustice Model

The synthesis of evidence related to the organizational injustice model is somewhat more problematic than in the previous two cases because the main systematic review did not include a meta-analysis of study findings with related forest plots (21). Moreover, this work included three different health outcomes, i.e., indicators of mental health, sickness absence data, and data on subjective well-being. The review identified prospective studies published between 1990 and 2012 with measures of organizational injustice as exposures and with data on one or several of the health outcomes mentioned. Having identified 403 studies from systematic literature search, the authors selected 11 studies that met all inclusion criteria. As the concept of organizational injustice is composed by four dimensions, these studies varied by the extent to which these dimensions were measured. Thus, relational injustice was assessed in ten of the 11 studies, procedural injustice in eight studies, and distributive injustice in three studies. Data on interactional injustice were not available. Here, we restrict the summary of results to studies that used mental health indicators as outcomes, assessed either as clinically validated depression or as questionnaire-based depressive symptoms.

Findings on associations of relational injustice with reduced mental health were retrieved from seven studies, with odds ratios ranging from 1.2 to 1.6. Importantly, in five of these studies, observed effects remained statistically significant after adjusting for the alternative work stress models of demand-control or effort-reward imbalance. One of the investigations analyzed change over time in organizational injustice and its association with change in mental health. Here, deterioration of injustice was related to an increased risk of reporting minor psychiatric morbidity. Related odds ratios were 1.81 (95% CI 1.48; 2.21) among men and 1.74 (95% CI 1.31; 2.37) among women (21). Procedural injustice was explored with regard to mental health in six studies, and five reported odds ratios, whereas one presented coefficients of path analysis. In all six studies, significant associations were observed, with odds ratios ranging from 1.4 to 1.9. In some cases, effects were adjusted for alternative models of work stress. Finally, one longitudinal study only tested the relationship of distributive injustice with mental health, using path analysis. Distributive justice was associated with reduced depressive symptoms after one year, adjusting for baseline level of these symptoms. A direct path was statistically significant, but no alternative work stress model was included in this analysis (21). The meta-review by Harvey et al. (30) also highlighted organizational justice as a relevant construct influencing mental health although the quality of the above systematic review was rated low.

Taken together, results support the notion that two components of organizational injustice, relational and procedural injustice, are associated with a moderately increased risk of poor mental health. However, the evidence base is less extensive than in previous cases, and the presentation of findings is limited due to missing meta-analysis and lack of inclusion of quality assessment. Following the publication of this systematic review, we identified four further recent publications on the topic. One study analyzed long-term sickness absence due to depression or anxiety disorders among Finnish public sector employees and observed a 25%–30% lower odds of sickness absence due to anxiety disorders, but not due to depression, among those experiencing interactional justice at work (41). In a second report, multiwave data on organizational justice and depressive symptoms were analyzed, where significant findings were mainly restricted to a protective effect of organizational justice among men who exhibited depressive symptoms (42). Third, in a Finnish cohort study on public employees, relational and procedural justice were associated with reduced risk of disability pension due to depression. However, after adjusting for the effects of effort-reward imbalance and demand-control, effects lost their statistical significance (43). Finally, a Danish prospective study assessed aggregated work-unit data of procedural and relational injustice at work, related to onset of new depression over a 2-year period (44). In this study of 4.237 public employees, the adjusted odds ratio of depression due to procedural injustice was 2.50 (95% CI 1.06; 5.88), and the odds ratio due to relational injustice was 3.14 (95% CI 1.37; 7.19). In summary, the above conclusion is strengthened further by integrating these recent findings.

## Discussion

In this report, we observed a moderately elevated risk of developing clinical depression or depressive symptoms following exposure to an adverse psychosocial work environment, as measured by three complementary theoretical models. Evidence was relatively strongest for demand-control,followed by effort-reward imbalance and organizational injustice. Each model represents a distinct perspective on work by emphasizing the job task profile, the employment contract, or the social relationships within organizations, respectively. Although statistical adjustment for alternative models was performed in a limited number of studies only, the reported findings offer three explanatory frameworks of the association under study. The current state of knowledge is nevertheless limited to some extent, as reports on health effects often relied on selected subcomponents rather than on a summary measure of the model. Moreover, implementation of a standard measurement of the underlying theoretical construct is desirable to advance cumulative knowledge.

An important conceptual and empirical question remains unanswered so far: How do these three models interact with risk of depression? Is there an additive or a cumulative effect if workers are exposed simultaneously to all three exposures? To our knowledge, one recent publication has tackled this problem. In a longitudinal study of disability pensions due to depression among 41.862 Finnish employees followed over a mean 3.1 years, the following results were obtained (45): Compared to the group without any exposure, those with comanifestation of all three exposures were 4.7 times more likely to experience a disability pension due to depression. The hazard ratio of 4.70 (95% CI 2.26; 7.79) was adjusted for a comprehensive set of confounding factors. Of interest, hazard ratios were 2.47 (job strain), 1.87 (effort-reward imbalance), and 1.66 (organizational injustice) if one exposure only was considered. This study calls for further research on clusters of work stress rather than single models, aiming at an identification of particularly “toxic” constellations of stressful work.

At the methodological level, future research is expected to clarify to what extent the observed prospective associations are representing a causal link. As mentioned, observational studies offer limited evidence on causality. This even holds true if the criteria supporting a causal relationship in epidemiologic research, as developed by Bradford Hill (46), are applied, such as consistency of results, strength of association, dose-response relationship, control of confounding and reverse causation, provision of data on mediating pathways, and reduction of effect following reduction of exposure.

The results of this review illustrate an impressive amount of consistency, as more than 40 prospective investigations reported positive findings of an association of stressful work, in terms of the three models, with depression, despite some heterogeneity of exposure measures. The strength of associations is moderate across all studies. This observation points to the multifactorial nature of depressive disorders, where it is obvious that socio-environmental exposures in general, and psychosocial work-related exposures in particular, provide a modest contribution only to the overall risk estimation of depression. For instance, in one study it was estimated that a population-attributable fraction of 14% of new cases of common mental disorders could have been theoretically prevented by eliminating high job strain (34). Dose-response as a further criterion of causality has not yet been analyzed to a sufficient extent. Studies with multiple exposure data over time or with different degrees of exposure intensity are required to this end. Up to now, most published studies offer data on one exposure assessment at baseline that has been linked to the subsequent probability of disease onset. Moreover, rigorous statistical approaches to multiwave data are required, such as a recently conducted fixed effects analysis, applying dynamic panel models with data on work stress and depressive symptoms from four waves (40).

Observed and unobserved confounding is considered a major challenge to epidemiological research in general. In case of our research question, substantial concern was devoted to the inclusion of relevant confounders in multivariable regression analyses, adjusting for their effects in a majority of studies. However, despite poor evidence, reverse causation cannot be excluded, specifically with regard to depression. This disorder often becomes manifest in early adulthood, and its recurrence at later stages is relatively frequent. Moreover, people’s increased mental vulnerability in earlier stages of their life course may shape occupational trajectories to some extent, putting them at higher risk of ending up in a less privileged work place (34).

The aetiology of depressive disorders is still not well known, and the same holds true for proposed stress-biological mechanisms acting as potential pathways from exposure to disease development. Altered functioning of the hypothalamic-pituitary-adrenal axis and increased endogenous inflammation were proposed as two promising markers of such pathways (8, 9). In fact, for all three models of an adverse psychosocial work environment, associations with altered cortisol secretion and/or increased inflammation were demonstrated (47–49). These preliminary data support a mediating role of stress-biological mechanisms linking exposure to the development of depressive disorders, although longitudinal evidence on these pathways is still missing. Finally, the criterion of health improvement as a result of exposure reduction has been dealt with in the frame of theory-based intervention studies. Again, for all three models of stressful work, company-based worksite - health promotion programs documented beneficial effects on mental health (50, 51). For instance, in three quasi-experimental intervention studies in Canada, the components of the demand-control and the effort-reward imbalance model were addressed by organizational changes, resulting in significant reductions of burn out or psychological distress. In one study, beneficial effects on burnout were manifest even 3 years after onset of intervention (52). Thus, the findings of these intervention studies point to the relevance of these theoretical models for targeted programs of primary and secondary prevention of mental disorders in occupational settings.

This discussion reveals substantial progress of research on psychosocial work-related exposures and risk of depression. In particular, conceptual and methodological developments in the frame of three theoretical models resulted in the generation of a substantial body of empirical evidence. At the same time, unresolved questions and challenges were identified that require further scientific inquiry.

### Limitations of this Review

This paper represents a meta-review perspective on a relevant body of recent research that already received a number of systematic reviews. Its aim was to assess strengths and weaknesses of current knowledge and to identify theoretical and methodological challenges that deserve further research. Pursuing this aim, nevertheless, results in several limitations. First, rather than following proposed recommendations for meta-synthesis data integration (30, 53), the report is restricted to a narrative presentation of its main messages. Second, we focused our review on three theoretical models, thus neglecting additional concepts of a health-adverse psychosocial work environment (e.g., “demand-resources” (54) or single psychosocial work-related factors (e.g., workplace bullying, 26), long working hours (55). The main reason for this selection was the availability of a cumulative body of empirical findings that reflects a significant interest of the research community in these theoretical approaches. Future reviews may represent a more comprehensive range of concepts and measures. For instance, the systematic review by Harvey et al. (30) proposed three integrative broad categories that include elements of all three models, termed imbalanced job design, occupational uncertainty, and lack of value and respect in the workplace. Third, as most studies discussed were conducted in high-income countries of the Western world, we do not justice to a globally relevant problem of work-related population health. There is an urgent need to extend this research to rapidly developing country. Finally, as one of the authors is the originator of one of the three models mentioned, there is a risk of reporting bias. However, every attempt was made to balance the presentation of findings. Importantly, this author was not involved in the systematic review on effort-reward imbalance and depression, the major source of reference in this respect.

### Implications for Practice

As a first practical implication, this new knowledge can be integrated into efforts of identifying people at risk for developing depression. This is an important extension of psychiatrists’ and psychotherapists’ task of recognizing and treating psychosocial risks among their patients. Several risk prediction assessments were developed, in line with the notion of the multifactorial nature of this disorder [e.g., (56)]. Specifying these assessments by including core information on adverse psychosocial work environments is an important further step towards targeting working people at risk. Once such assessments have been established in organizations and businesses, occupational physicians, and other health professionals can use this information, observing strict data protection policies. Based on available information, the development and implementation of measures of primary prevention provides an important second practical implication. This implementation has to take into account available, specifically qualified personnel and appropriate settings of program delivery. Several such programs were already successfully applied with the aim of strengthening employed people’s resources of coping with stress at work (57). They usually contain nonspecific elements, such as relaxation, meditation, anger management, or self-assertiveness. More specific measures, derived from the abovementioned work stress models, include group discussions on the reorganization of work tasks and schedules, supported team meetings with managers to improve communication, leadership and appreciation, or the preparation of workshops devoted to the elaboration of career and skill development (52, 58).

A third practical implication concerns secondary prevention, i.e., the improvement of return to work following treated manifest depression. As health-promoting psychosocial work environments were shown to increase rates of return to work in the chronically ill, structural and interpersonal measures of work-related stress reduction may exert favorable effects on depressive patients who are able and motivated to return to work.

### Conclusion

In conclusion, this review documents substantial progress of research on psychosocial work-related exposures and risk of depression. In particular, conceptual and methodological developments in the frame of the three theoretical models demand-control, effort-reward imbalance, and organizational injustice resulted in the generation of a substantial body of new empirical evidence. The robustness of findings calls for their recognition by psychiatrists and psychotherapists in diagnosing and treating patients. Moreover, they can instruct measures and programs of primary and secondary prevention of depressive disorders. At the same time, unresolved questions and challenges were identified that require further scientific inquiry.

## Author Contributions

JS conceptualized and wrote the manuscript. NW contributed by systematic literature search and by critically commenting and improving the final version of the manuscript.

## Funding

This work was supported by a senior professorship awarded to JS by the faculty of medicine, Heinrich-Heine University of Düsseldorf, Germany.

## Conflict of Interest

The authors declare that the research was conducted in the absence of any commercial or financial relationships that could be construed as a potential conflict of interest.
